# Circulating exosomal long non‐coding RNAs in patients with acute myocardial infarction

**DOI:** 10.1111/jcmm.15589

**Published:** 2020-07-10

**Authors:** Mei‐Li Zheng, Xiao‐Yan Liu, Rui‐Juan Han, Wen Yuan, Kai Sun, Jiu‐Chang Zhong, Xin‐Chun Yang

**Affiliations:** ^1^ Heart Center Beijing Chao‐Yang Hospital Capital Medical University Beijing China; ^2^ Beijing Key Laboratory of Hypertension Research Beijing Chao‐Yang Hospital Capital Medical University Beijing China; ^3^ Medical Research Center Beijing Chao‐Yang Hospital Capital Medical University Beijing China; ^4^ Department of Radiology State Key Laboratory of Cardiovascular Disease Fu Wai Hospital National Center for Cardiovascular Diseases Chinese Academy of Medical Sciences and Peking Union Medical College Beijing China; ^5^ Department of Radiology Fuwai Hospital Chinese Academy of Medical Sciences Shenzhen China

**Keywords:** acute myocardial infarction, biomarkers, exosomal lncRNAs, heart failure, prognosis

## Abstract

Exosomes are attracting considerable interest in the cardiovascular field as the wide range of their functions is recognized in acute myocardial infarction (AMI). However, the regulatory role of exosomal long non‐coding RNAs (lncRNAs) in AMI remains largely unclear. Exosomes were isolated from the plasma of AMI patients and controls, and the sequencing profiles and twice qRT‐PCR validations of exosomal lncRNAs were performed. A total of 518 differentially expressed lncRNAs were detected over two‐fold change, and 6 kinds of lncRNAs were strikingly elevated in AMI patients with top fold change and were selected to perform subsequent validation. In the two validations, lncRNAs ENST00000556899.1 and ENST00000575985.1 were significantly up‐regulated in AMI patients compared with controls. ROC curve analysis revealed that circulating exosomal lncRNAs ENST00000556899.1 and ENST00000575985.1 yielded the area under the curve values of 0.661 and 0.751 for AMI, respectively. Moreover, ENST00000575985.1 showed more significant relationship with clinical parameters, including inflammatory biomarkers, prognostic indicators and myocardial damage markers. Multivariate logistic model exhibited positive association of ENST00000575985.1 with the risk of heart failure in AMI patients. In summary, our data demonstrated that circulating exosomal lncRNAs ENST00000556899.1 and ENST00000575985.1 are elevated in patients with AMI, functioning as potential biomarkers for predicting the prognosis of pateints with AMI.

## 1 INTRODUCTION

Acute myocardial infarction (AMI) is a major cause of morbidity and mortality worldwide,^1^ resulting in sudden myocardial tissue ischaemia, and sudden cardiac death.^2^ Thus, it is necessary to identify novel biomarkers for early diagnosis and prognosis prediction of AMI, as these may assist in providing valuable therapies.

Long non‐coding RNAs (lncRNAs) are defined as non–protein‐coding transcripts that are longer than 200 nucleotides. An increasing body of evidence suggested that lncRNAs play biologically fundamental roles in the occurrence and development of cardiovascular diseases.^3^, ^4^, ^5^ LncRNAs are differently expressed and have been shown to be used as biomarkers for the progression of coronary artery disease, atrial fibrillation and heart failure.^6^, ^7^, ^8^ In addition, lncRNAs might act as biomarkers in the early diagnosis of AMI and also help in predicting its outcome.^9^, ^10^, ^11^ Compared with lncRNAs present in extracellular fluids of the body, those lncRNAs that are packaged in exosomes are highly stable. Exosomal lncRNAs have lipid bilayers and could protect them from enzymatic degradation of RNA enzymes in bodily fluids. Thus, exosomal lncRNAs have a relatively long and stable duration of expression in the cardiovascular system. Previous studies have shown that exosomal lncRNAs were involved in various diseases and can be used as biomarkers.^12^, ^13^, ^14^ However, few reports have focused on the role of exosomal lncRNAs in AMI. Hence, in the present study, we investigated the role of circulating exosomal lncRNAs in AMI patients and provide some potential biomarkers for diagnosis and prognosis prediction of AMI.

## 2 STUDY PATIENTS AND METHODS

### 2.1 Study patients

The present study compared the sequencing profiles of circulating exosomal lncRNA in AMI patients (n = 15) with controls (n = 15). This was first validated in 20 AMI patients and 20 controls, and then second validation in 85 AMI patients and 48 controls. For sequencing profiles, the blood samples of every 5 AMI patients as well as the controls were pooled into one sample, and thus, ‘3 AMI’ and ‘3 control’ samples were profiled. All AMI patients enrolled in the present study had a heart attack within 12 hours from the time of admission, and revascularization was successfully performed in the emergency department before hospitalization.

AMI was defined based on clinical symptoms, typical changes in electrocardiogram (ECG), elevated cardiac biomarkers troponin‐I (TnI) and creatine kinase MB (CKMB) given by the Universal Definition of myocardial infarction.^15^ The controls included were of non‐coronary chest pain patients (NCCP), that had chest pain, normal cardiac biomarkers and most importantly, no coronary stenosis as confirmed by angiography.

All patients were recruited from Beijing Chao‐Yang Hospital Affiliated to Capital Medical University in China. Written informed consent was obtained and signed from all participants. This study was conducted in accordance with the Declaration of Helsinki, and the research protocol was approved by the Ethics Committee of Beijing Chao‐Yang Hospital.

### 2.2 Exosomal Isolation and Identification

The exosomes were isolated using a commercial kit (Qiagen Inc), following the manufacturer's instructions, and identified by transmission electron microscopy (TEM), nanoparticle tracking analysis (NTA) and Western blotting. Isolated exosomes were examined by TEM and NTA as described previously.^16^, ^17^ Western blotting of exosomal marker protein CD63 (Abcam) and heat shock protein 70 (HSP70) (Abcam) was done as previously described.^18^

### 2.3 Exosomal RNA Extraction, RNA Sequencing and Quantitative RT‐PCR

The lncRNAs were extracted from exosomes by using a commercial kit (Qiagen Inc) according to the manufacturer's protocol. RNA sequencing procedure was done as described in the methods section of our previous study.^19^ The relative expression levels of lncRNA were quantified using ViiA 7 Real‐Time PCR System (Applied Biosystems) according to standard methods, and the forward and reverse primers included were listed in Table S1. The lncRNA IDs were searched in Ensembl Human GRCh37.p13.

### 2.4 Laboratory measurements and echocardiography

Fasting venous blood samples were taken within the first 24 hours of admission. Blood samples for isolating the exosomes were collected by venipuncture into EDTA containing tubes. All patients received laboratory measurements including lipids, glucose, creatinine and other items. Echocardiography was performed within 72 hours after admission. The items measured included left ventricular end‐diastolic diameter (LVEDD), left ventricular ejection fraction (LVEF) and others.

### 2.5 Clinical conditions

Heart failure was defined by NT‐BNP > 1000 pg/mL, or LVEDD > 55 mm, or LVEF < 40%. Major adverse cardiovascular events (MACE) included cardiac death, ventricular fibrillation and cardiac shock. Long‐term hospitalized was defined as hospital time > 5 days.

### 2.6 Statistical analysis

All analyses were performed using SPSS 24.0 software (IBM). Continuous variables with normal distribution were expressed as mean ± standard deviation (SD) and compared by two‐sample *t* test, while those with non‐normal distributed were expressed as quartiles and compared by Mann‐Whitney *U* test. Categorical variables were expressed as percentages and numbers, and compared using the chi‐square test. Spearman's correlation coefficients were used to assess the relationships between variables. Receiver operating characteristic curves and areas under the curve (AUCs) were computed. All statistical tests were two‐tailed, and *P*‐values of .05 were considered to be statistically significant.

## 3 RESULTS

### 3.1 Sequencing profiles of circulating exosomal lncRNA in AMI patients

The clinical characteristics of sequencing samples are provided in Table S2. There were mostly well balanced between AMI patients and controls, except for the higher fasting glucose level in AMI patients. Given that the number of diabetics was the same in the two groups, the higher fasting glucose level in AMI patients was considered to be caused by stressed hyperglycaemia.

**Figure 1:**
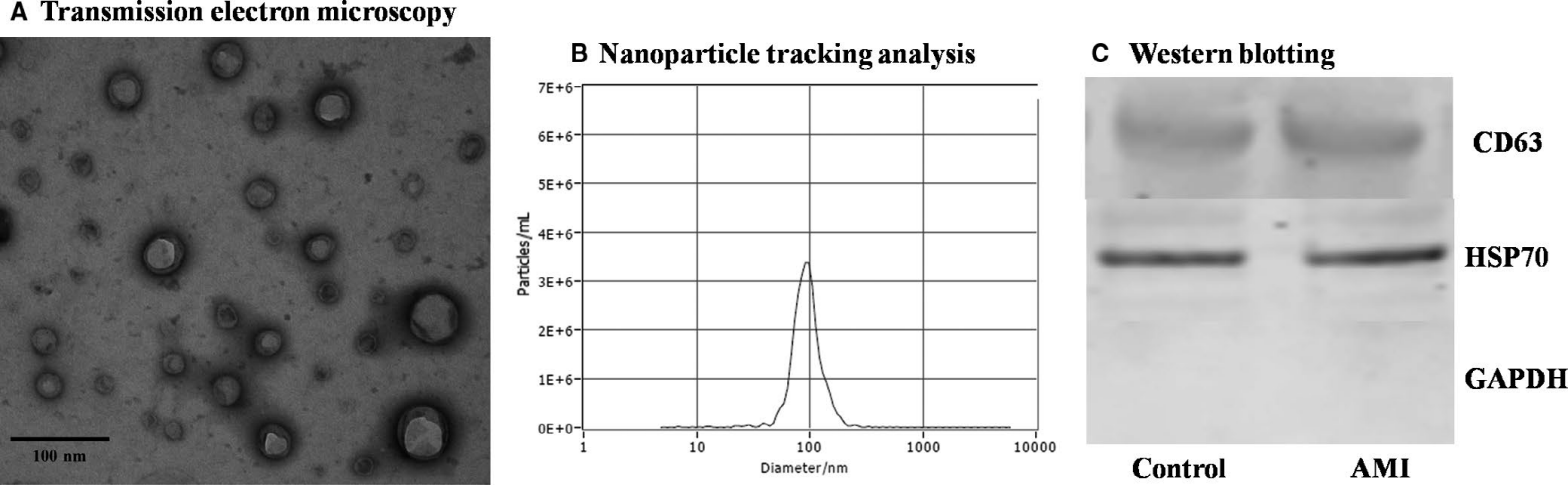
Exosome identification for sequencing plasma samples by TEM (A), nanoparticle tracking analysis (B) and Western blotting (C), respectively. TEM: transmission electron microscopy; AMI, acute myocardial infarction; and HSP70, heat shock protein 70

Exosomes from next‐generation sequencing of plasma samples were characterized by TEM, nanoparticle tracking analysis and Western blotting. TEM analysis (Figure 1A) revealed that the exosomes had circular‐like, bilayer membrane vesicle structure and were around 100 nm in diameter according to nanoparticle tracking analysis (Figure 1B). Western blot analysed the expression of specific proteins CD63 and HSP70 (Figure 1C), indicating exosomal marker protein in plasma samples.

**Figure 2:**
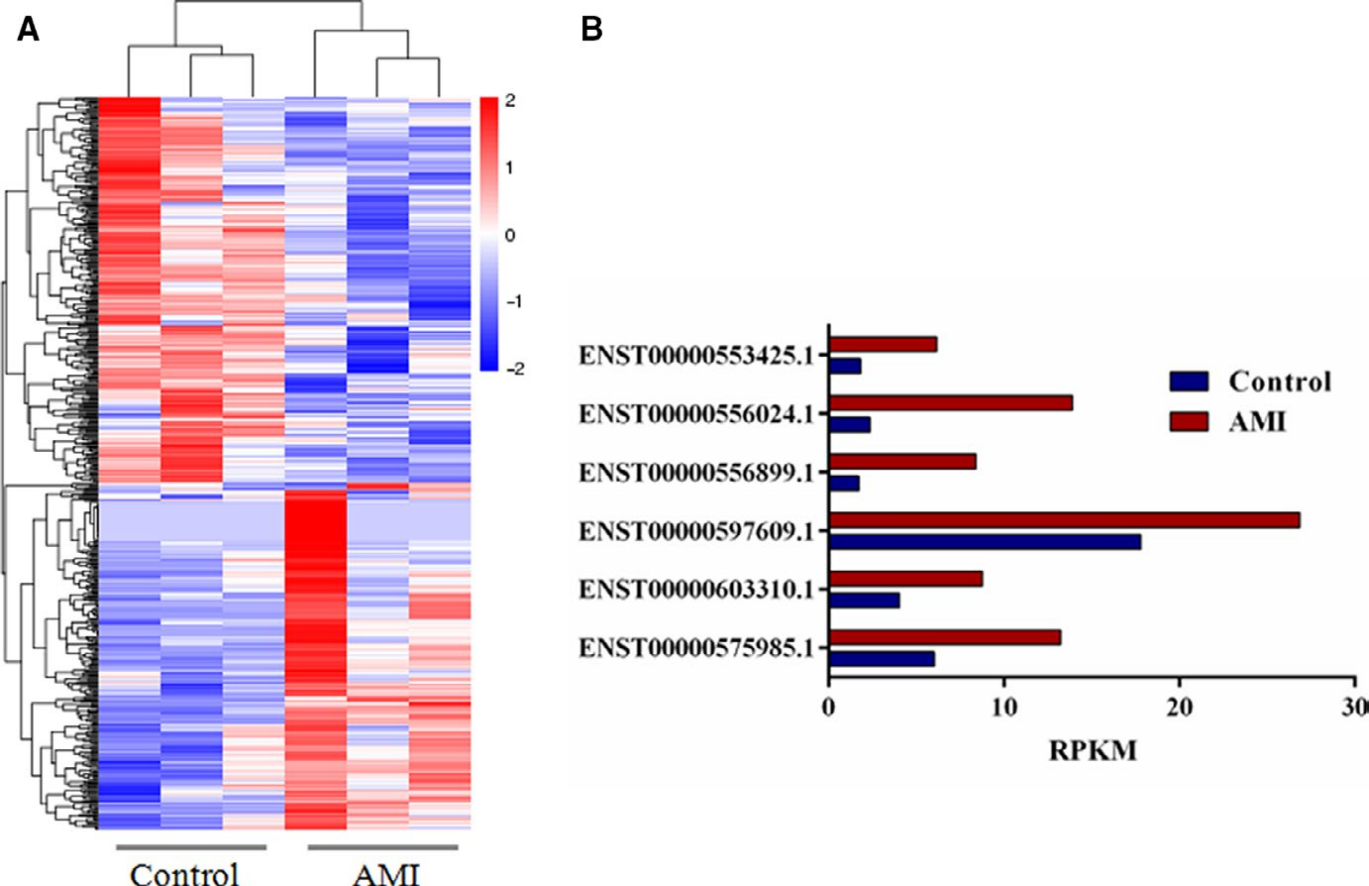
Circulating exosomal lncRNA sequencing in AMI. A: the hierarchical clustering of substantially differentially expressed (*P* < .05; fold change > 2) lncRNAs in control and AMI; B: Six up‐regulated lncRNAs in AMI were selected for the first validation. AMI, acute myocardial infarction

Using a twofold expression difference as a cut‐off, a total of 518 differentially expressed lncRNAs were detected in the plasma exosomes of the two groups with 245 up‐regulated lncRNAs and 273 down‐regulated lncRNAs (*P* < .05; Figure 2A). From the 245 up‐regulated lncRNAs identified, six highly expressed lncRNAs with top fold change were selected for the first validation (Figure 2B).

### 3.2 First validation of circulating exosomal lncRNA

**Figure 3:**
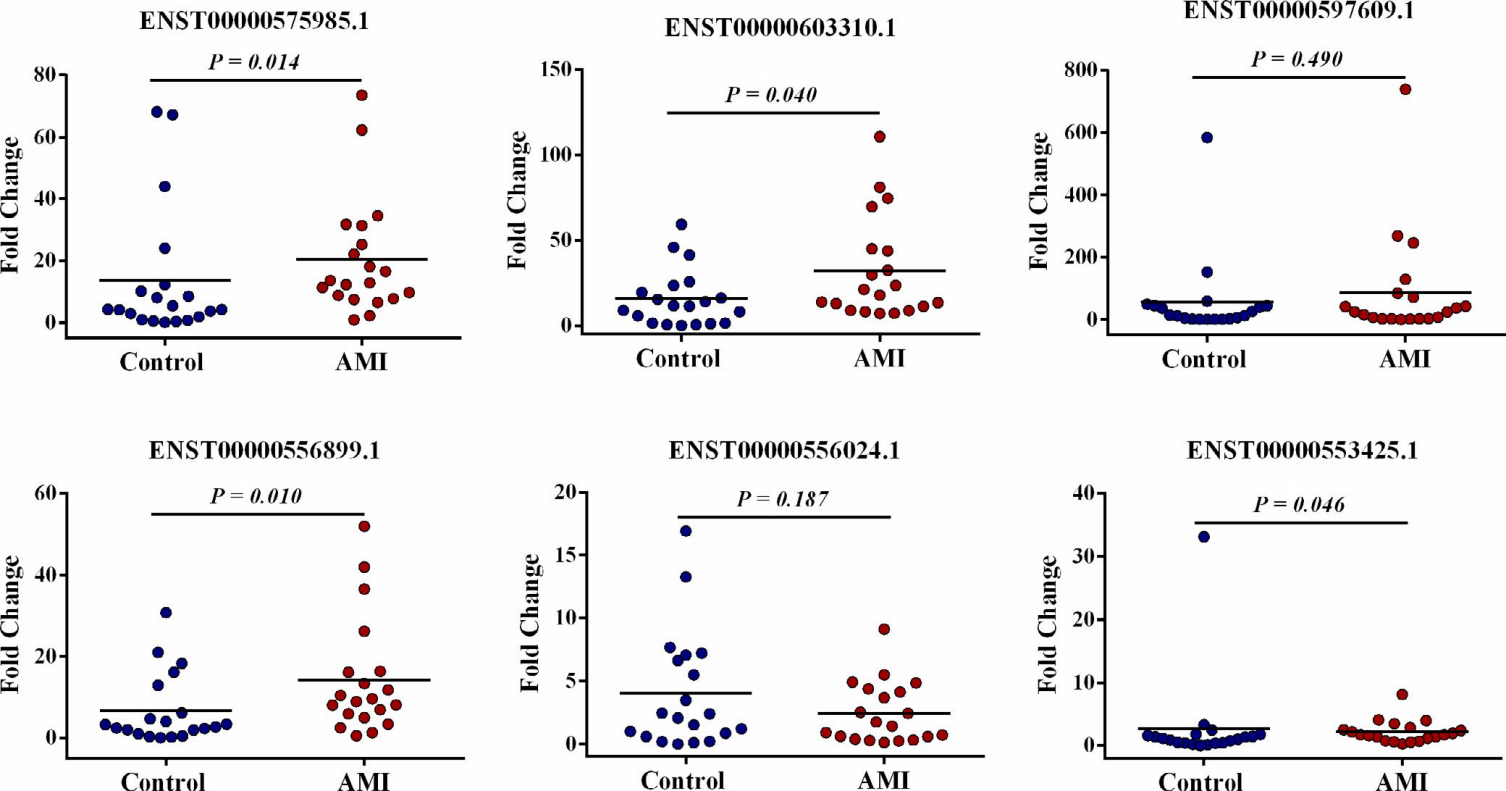
qRT‐PCR analysis of expression of 6 kinds of circulating exosomal lncRNAs (ENST00000553425.1, ENST00000556024.1, ENST00000556899.1, ENST00000597609.1, ENST00000603310.1 and ENST00000575985.1) were selected in the first validation

The first validation was performed in 20 AMI patients and 20 controls, and their clinical characteristics are shown in Table 1. The results showed no differences between the two groups in age, physical data (including heart rate [HR], systolic blood pressure [SBP], diastolic blood pressure [DBP], body mass index [BMI]), historical data (including history of hypertension and diabetes, and family history) and living habits (including smoking and drinking). The circulating exosomal lncRNA ENST00000553425.1, lncRNA ENST00000556024.1, lncRNA ENST00000556899.1, lncRNA ENST00000597609.1, lncRNA ENST00000603310.1 and lncRNA ENST00000575985.1 were selected, which were highly expressed in both groups with top fold change, to perform the first validation, while lncRNA ENST00000553425.1 (*P* = .046, fold change = 1.60), lncRNA ENST00000556899.1 (*P* = .010, fold change = 3.13), lncRNA ENST00000603310.1 (*P* = .040, fold change = 1.69) and lncRNA ENST00000575985.1 (*P* = .014, fold change = 3.11) showed significant up‐regulation in AMI patients in the first validation (Figure 3). Using a twofold expression difference as a cut‐off, lncRNA ENST00000556899.1 and lncRNA ENST00000575985.1 were selected for subsequent second validation.

**Table 1:** Clinical characteristics of patients in the first and second validations in controls and patients with AMI

	First validation			Second validation		
	Control (N = 20)	AMI (N = 20)	*P* value	Control (N = 48)	AMI (N = 85)	*P* value
Age, y	55.9 ± 11.1	59.8 ± 15.0	.356	56.6 ± 10.8	59.4 ± 10.7	.155
Male, n (%)	9 (45.0)	17 (85.0)	.008	27 (56.3)	77 (90.6)	<.001
Current smoker, n (%)	8 (40.0)	9 (45.0)	.530	4 (8.3)	57 (67.1)	<.001
Current drinker, n (%)	3 (15.0)	8 (40.0)	.077	11 (22.9)	25 (29.4)	.271
Physical data						
Heart rate, beats/min	72.0 [65.0‐73.0]	72.5 [65.3‐78.8]	.327	71.0 [63.8‐79.8]	76.0 [67.0‐87.0]	.037
Systolic blood pressure, mm Hg	129.5 [119.5‐136.0]	122.0 [110.0‐132.3]	.149	128.5 [120.3‐140.0]	121.0 [112.5‐136.5]	.081
Diastolic blood pressure, mm Hg	72.5 [66.3‐81.3]	68.5 [58.5‐80.5]	.165	77.5 [70.0‐84.8]	74.0 [66.5‐81.5]	.429
Body mass index, kg/m^2^	24.7 ± 4.1	25.2 ± 1.0	.552	26.1 ± 3.5	25.3 ± 2.6	.135
Historical data						
Hypertension, n (%)	7 (35.0)	10 (50.0)	.337	19 (39.6)	48 (56.5)	.061
Diabetes, n (%)	0 (0.0)	2 (10.0)	.468	5 (10.4)	20 (23.5)	.063
Family history, n (%)	3 (15.0)	6 (30.0)	.449	13 (27.1)	20 (23.5)	.649
Laboratory data						
C‐reactive protein, mg/L	1.51 [0.82‐2.24]	2.86 [1.34‐11.54]	.026	1.10 [0.59‐2.57]	6.46 [2.44‐13.00]	<.001
ESR, mm/h	3.00 [2.00‐6.00]	4.50 [2.00‐10.50]	.497	5.00 [2.00‐10.50]	6.00 [3.00‐13.00]	.186
Leukocyte, ×10^9^/L	5.95 [5.46‐7.17]	8.55 [6.67‐11.40]	.003	5.90 [5.33‐7.25]	9.34 [7.03‐11.51]	<.001
Neutrophil, ×10^9^/L	3.40 [2.89‐4.25]	6.03 [4.07‐9.09]	<.001	3.73 [3.12‐4.52]	7.12 [4.95‐10.34]	<.001
Lymphocyte, ×10^9^/L	2.00 [1.65‐2.30]	1.47 [0.98‐1.68]	<.001	1.67 [1.47‐2.07]	1.60 [1.00‐2.25]	.19
Hemoglobin, g/L	124 0.0 [117.5‐137.0]	137.0 [121.3‐145.0]	.114	134.5 [124.3‐147.8]	141.0 [130.0‐156.0]	.026
Platelets, ×10^9^/L	246.0 [209.5‐266.5]	212.0 [179.5‐268.5]	.253	213.0 [185.5‐259.0]	200.0 [167.0‐244.5]	.304
AST, U/L	18.0 [16.0‐22.8]	61.5 [24.0‐214.0]	<.001	19.0 [17.0‐22.5]	43.0 [26.0‐146.5]	<.001
ALT, U/L	15.5 [12.0‐23.3]	28.5 [17.3‐48.0]	.007	16.0 [12.0‐21.5]	30.0 [19.5‐47.0]	<.001
Total cholesterol, mmol/L	4.28 [3.53‐4.42]	4.59 [4.21‐5.13]	.091	4.22 [3.58‐5.18]	4.38 [3.50‐5.31]	.785
HDL‐C, mmol/L	1.15 [0.93‐1.58]	1.00 [0.90‐1.38]	.398	1.10 [0.95‐1.30]	1.05 [0.80‐1.24]	.114
LDL‐C, mmol/L	2.25 [1.90‐2.73]	2.85 [2.43‐3.28]	.015	2.70 [1.95‐3.20]	2.57 [2.00‐3.51]	.548
Triglycerides, mmol/L	1.28 [0.79‐1.75]	1.44 [0.83‐1.81]	.602	1.25 [0.99‐1.80]	1.51 [1.07‐2.45]	.018
Fast glucose, mmol/L	5.06 [4.62‐5.32]	5.56 [5.08‐8.18]	.024	4.83 [4.35‐5.22]	6.57 [5.42‐8.45]	<.001
HbA1C, %	5.60 [5.28‐5.93]	5.70 [5.50‐6.90]	.271	5.65 [5.38‐5.93]	6.00 [5.60‐7.30]	<.001
BUN, mmol/L	4.14 [3.70‐5.64]	5.47 [4.19‐6.46]	.068	5.16 [4.68‐5.92]	5.12 [4.52‐6.56]	.566
Serum creatinine, μmol/L	62.8 [53.3‐72.6]	68.5 [61.4‐81.8]	.063	65.6 [57.8‐74.4]	74.6 [66.0‐81.1]	.001
Uric acid, μmol/L	350.0 [292.5‐396.3]	322.0 [294.0‐393.0]	.583	331.5 [271.0‐373.0]	365.0 [297.0‐449.0]	.022
Na+, mmol/L	141.5 [139.9‐142.7]	139.8 [138.5‐141.0]	.021	142.0 [140.1‐142.6]	139.9 [137.9‐142.6]	.003
K+, mmol/L	4.00 [3.73‐4.25]	4.20 [3.85‐4.40]	.035	3.80 [3.70‐4.10]	4.10 [3.80‐4.35]	.012
Serum albumin, g/L	39.5 [38.1‐43.2]	40.1 [37.0‐41.7]	.718	39.4 [36.9‐41.4]	40.2 [38.1‐42.1]	.104
Free triiodothyronine, pg/mL	2.79 [2.64‐3.01]	2.52 [2.35‐2.79]	.008	2.95 [2.70‐3.12]	2.55 [2.27‐2.77]	<.001
Free tetraiodothyronine, ng/dL	1.06 [0.98‐1.16]	1.18 [1.07‐1.31]	.035	1.12 [1.02‐1.25]	1.14 [1.07‐1.24]	.59
sTSH, uIU/mL	1.85 [1.12‐2.41]	1.13 [0.65‐1.45]	.006	2.05 [1.25‐3.25]	1.17 [0.62‐1.62]	<.001
Troponin‐I, ng/mL	0.00 [0.00‐0.01]	12.38 [2.52‐71.95]	<.001	0.00 [0.00‐0.02]	6.56 [0.39‐31.77]	<.001
CKMB, ng/Ml	0.60 [0.01‐1.25]	15.05 [1.00‐105.48]	.001	0.50 [0.10‐0.80]	14.00 [2.90‐49.35]	<.001
Fibrinogen, mg/dL	254.0 [237.5‐293.5]	280.0 [241.2‐333.9]	.374	240.3 [206.4‐299.4]	283.1 [257.0‐325.8]	<.001
Medicine use						
Aspirin, n (%)	6 (30.0)	20 (100.0)	<.001	24 (50.0)	76 (89.4)	<.001
Clopidogrel, n (%)	3 (15.0)	20 (100.0)	<.001	4 (8.3)	65 (76.5)	<.001
Ticagrelor, n (%)	0 (0.0)	0 (0.0)	1.000	0 (0.0)	16 (18.8)	.001
ACEI/ARB, n (%)	4 (20.0)	6 (30.0)	.465	10 (20.8)	53 (62.4)	<.001
Statin, n (%)	12 (60.0)	20 (100.0)	.006	33 (68.8)	76 (89.4)	.003
β‐blocker	4 (20.0)	11 (55.0)	.022	14 (29.2)	60 (70.6)	<.001
CCB, n (%)	6 (30.0)	1 (5.0)	.096	11 (22.9)	8 (9.4)	.033
Prognostic data						
LVEDD, mm	45.0 [44.0‐49.0]	46.0 [44.3‐49.8]	.478	46.0 [43.0‐50.0]	48.5 [46.0‐50.8]	.001
LVEF, %	71.0 [66.5‐72.5]	65.5 [59.3‐73.8]	.069	67.0 [66.0‐73.0]	55.0 [50.0‐65.0]	<.001
NT‐BNP, pg/mL	54.4 [16.0‐109.9]	760.3 [417.6‐1171.0]	.002	45.1 [26.7‐89.3]	311.1 [176.1‐869.9]	<.001
Cardiac shock, n (%)	0 (0.0)	0 (0.0)	1.000	0 (0.0)	3 (3.5)	.479
Malignant arrhythmia, n (%)	0 (0.0)	2 (10.0)	.468	0 (0.0)	4 (4.7)	.319
Death, n (%)	0 (0.0)	0 (0.0)	1.000	0 (0.0)	1 (1.2)	.771
Hospital time, d	3.50 [3.00‐6.00]	7.50 [6.00‐9.00]	<.001	4.00 [3.00‐6.00]	11.00 [6.00‐15.00]	<.001

Abbreviations: ACEI/ARB, angiotensin‐converting enzyme inhibitors/angiotensin receptor blockers; ALT, alanine aminotransferase; AMI, acute myocardial infarction; AST, aspartate aminotransferase; BUN, blood urea nitrogen; CCB, calcium antagonist; CKMB, creatine kinase MB; ESR, erythrocyte sedimentation rate; HbA1C, glycosylated haemoglobin; HDL‐C, high density lipoprotein cholesterol; LDL‐C, low density lipoprotein cholesterol; LVEDD, left ventricular end‐diastolic diameter; LVEF, left ventricular ejection fraction; NT‐BNP, N‐terminal proB‐type natriuretic peptide; sTSH, thyroid‐stimulating hormone.

### 3.3 Second validation of circulating exosomal lncRNA

**Figure 4:**
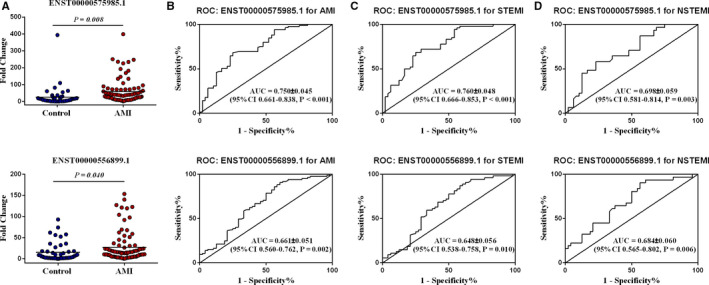
Circulating exosomal lncRNA ENST00000575985.1 and ENST00000556899.1 in the second validation. A, qRT‐PCR analysis of expression of ENST00000575985.1 and ENST00000556899.1; B, receiver operating characteristic (ROC) curves analyses of ENST00000575985.1 and ENST00000556899.1 for all AMI; C: ROC curves analyses for STEMI; D: ROC curves analyses for NSTEMI. AMI, acute myocardial infarction; STEMI, ST‐segment elevation myocardial infarction; and NSTEMI, non–ST‐segment elevation myocardial infarction

The second validation was performed in 85 AMI patients and 48 controls, and their clinical characteristics are shown in Table 1. Their demographic characteristics were mostly well balanced between the two groups. In the second validation, lncRNA ENST00000556899.1 (*P* = .040, fold change = 2.49) (Figure 4A) and lncRNA ENST00000575985.1 (*P* = .008, fold change = 3.14) (Figure 4A) were shown to be significantly up‐regulated in AMI patients when compared with controls. Receiver operating characteristic (ROC) curve analysis of circulating exosomal lncRNA ENST00000556899.1 showed an AUC = 0.661 ± 0.051 (95% CI 0.560‐0.762, *P* = .002) for all AMI patients (Figure 4B); AUC = 0.648 ± 0.056 (95% CI 0.538‐0.758, *P* = .010) for ST‐segment elevation myocardial infarction (STEMI) patients (Figure 4C); and AUC = 0.684 ± 0.060 (95% CI 0.565‐0.802, *P* = .006) for non‐ST‐segment elevation myocardial infarction (NSTEMI) patients (Figure 4D). ROC curve analysis of circulating exosomal lncRNA ENST00000575985.1 showed an AUC = 0.751 ± 0.045 (95% CI 0.661‐0.838, *P* < .001) for all AMI patients (Figure 4B); AUC = 0.760 ± 0.048 (95% CI 0.666‐0.853, *P* < .001) for STEMI patients (Figure 4C); and AUC = 0.698 ± 0.059 (95% CI 0.581‐0.814, *P* = .003) for NSTEMI patients (Figure 4D).

Association between circulating exosomal lncRNAs and clinical parameters.

Circulating exosomal lncRNA ENST00000575985.1 showed more significant relationships with clinical parameters when compared with lncRNA ENST00000556899.1. LncRNA ENST00000575985.1 showed association with inflammatory biomarkers, including C‐reactive protein (CRP) (*r* = .357, *P* < .001), erythrocyte sedimentation rate (ESR) (*r* = .216, *P* = .037), leukocyte count (*r* = .228, *P* = .008) and neutrophil count (*r* = .207, *P* = .017), and also showed correlation with prognostic indicators, including LVEDD (*r* = .243, *P* = .008), LVEF (*r* = −.358, *P* < .001), NT‐BNP (*r* = .305, *P* = .002) and hospital time (*r* = .287, *P* = .001). Both of the two lncRNAs were associated with myocardial damage markers, including TNI (for lncRNA ENST00000575985.1, *r* = .369, *P* < .001; for lncRNA ENST00000556899.1, *r* = .313, *P* < .001), CKMB (for lncRNA ENST00000575985.1, *r* = .222, *P* = .010; for lncRNA ENST00000556899.1, *r* = .213, *P* = .014) and aspartate aminotransferase (AST) (for lncRNA ENST00000575985.1, *r* = .265, *P* = .002; for lncRNA ENST00000556899.1, *r* = .211, *P* = .016) (Table 2). In multivariate logistic model, after adjusting for age, gender, BMI, hypertension and diabetes history, smoking and drinking habits, the circulating exosomal lncRNA ENST00000575985.1 was positively associated with the risk of severe heart failure in AMI patients (OR = 1.046, 95%CI 1.005‐1.089, *P* = .029) (Table 3).

**Table 2:** Associations between circulating exosomal lncRNA ENST00000575985.1 and ENST00000556899.1 with clinical parameters

	lncRNA ENST00000575985.1		lncRNA ENST00000556899.1	
	*r*	*P* value	*r*	*P* value
Age	.018	.835	.02	.822
Hospital time	**.287**	**.001**	.108	.217
LVEDD	**.243**	**.008**	.136	.14
LVEF	**−.358**	**<.001**	−.106	.251
Heart rate	**.198**	**.023**	.041	.64
Systolic blood pressure	**−.185**	**.033**	−.065	.457
Diastolic blood pressure	−.075	.388	.048	.582
Body mass index	**−.209**	**.042**	−.066	.528
C‐reactive protein	**.357**	**<.001**	.089	.347
ESR	**.216**	**.037**	.065	.534
Leukocyte	**.228**	**.008**	.057	.516
Neutrophil	**.207**	**.017**	.157	.072
Lymphocyte	−.102	.243	−.039	.659
Hemoglobin	.081	.352	.037	.674
Platelets	.028	.745	**−.182**	**.036**
AST	**.265**	**.002**	**.211**	**.016**
ALT	**.285**	**.001**	.17	.052
Total cholesterol	.114	.193	.099	.261
HDL‐C	**−.193**	**.03**	**−.184**	**.038**
LDL‐C	.125	.16	.035	.693
Triglycerides	.13	.146	.026	.772
Fast glucose	**.273**	.002	.052	.564
HbA1C	.175	.094	.105	.318
BUN	.068	.442	−.006	.946
Serum creatinine	.158	.071	**.214**	**.014**
Uric acid	.172	.092	.117	.252
Na+	−.12	.171	.011	.899
K+	.146	.097	−.028	.75
Serum albumin	.031	.725	−.041	.644
Free triiodothyronine	−.148	.164	−.084	.433
Free tetraiodothyronine	−.031	.77	.022	.839
sTSH	−.171	.109	−.161	.131
Troponin‐I	**.369**	**<.001**	**.313**	**<.001**
CKMB	**.222**	**.010**	**.213**	**.014**
Fibrinogen	**.178**	**.044**	.004	.961
NT‐BNP	**.305**	**.002**	**.334**	**.001**

Abbreviations: ALT, alanine aminotransferase; and sTSH, thyroid‐stimulating hormone; AST, aspartate aminotransferase; BUN, blood urea nitrogen; CKMB, creatine kinase MB; ESR, erythrocyte sedimentation rate; HbA1C, glycosylated haemoglobin; HDL‐C, high density lipoprotein cholesterol; LDL‐C, low density lipoprotein cholesterol; LVEDD, left ventricular end‐diastolic diameter; LVEF, left ventricular ejection fraction; NT‐BNP, N‐terminal proB‐type natriuretic peptide. Bold indicates statistical significant value.

**Table 3:** Multivariate logistic analysis of circulating exosomal lncRNAs in AMI patients^a^

	ENST00000575985.1			ENST00000556899.1		
	OR	95%CI	*P* value	OR	95%CI	*P* value
Heart failure	**1.046**	**1.005‐1.089**	**.029**	0.989	0.963‐1.016	.414
long‐term hospitalized	1.022	0.956‐1.093	.521	1.002	0.970‐1.036	.890
MACE	0.983	0.928‐1.042	.572	0.958	0.877‐1.047	.345

Abbreviations: AMI, acute myocardial infarction; MACE, major adverse cardiovascular events. Bold indicates statistical significant value.

^a^ The model was adjusting for age, gender, BMI, hypertension and diabetes history, smoking and drinking habits.

## 4 DISCUSSION

Extracellular vesicles (EVs)—particularly exosomes and microvesicles (MVs)—are attracting considerable interest in the cardiovascular field as the wide range of their functions is recognized in various cardiovascular diseases, including ischaemic heart disorders.^20^ These capabilities contain transporting regulatory molecules including different RNA species, lipids and proteins through the extracellular space including blood and delivering these cargos to recipient cells to modify cellular activity.^20^ EVs powerfully stimulate angiogenesis and may protect the heart against AMI. The present study revealed some interesting findings, in which the circulating exosomal lncRNAs might act as potential biomarkers for patients with AMI, especially the lncRNA ENST00000575985.1, and the lncRNA ENST00000575985.1 might be associated with the prognosis of AMI.

Exosomes as main EVs are around 100 nm in diameter and contain various informational particles, such as lncRNAs, miRNAs, mRNAs and proteins.^20^ Notably, exosomes are easily accessible in the extracellular fluids of the body, which have been shown to be as potential biomarkers in heart diseases.^20^, ^21^, ^22^ Previous studies have demonstrated that exosomal ncRNAs could serve as diagnostic biomarkers in various tumour and cardiovascular diseases.^20^, ^22^, ^23^, ^24^ EVs of different sources may be useful biomarkers of cardiovascular disease identities such as AMI and heart failure.^20^ Serum exosomal miRNA‐320d is a promising non‐invasive diagnostic biomarker for distinguishing metastatic from non‐metastatic colorectal cancer (CRC).^23^ Besides, the combination of miR‐320d and carcinoembryonic antigen had an AUC of 0.804 for the diagnosis of patients with metastatic CRC.^23^ Additionally, serum exosomal lncRNA MIAT levels were significantly higher in gastric cancer (GC) patients than in gastric adenoma patients and healthy controls.^24^ Interestingly, gastric adenoma patients with higher serum exosomal MIAT expression were more prone to develop GC,^24^ indicating serum exosomal lncRNA MIAT might serve as a promising novel biomarker for monitoring the progression of GC. Exosomal miRNA might act as diagnostic biomarkers in AMI and play an important role in the pathophysiology of AMI.^25^, ^26^ However, there is no evidence regarding the exact role of exosomal lncRNAs in AMI. The lncRNAs present in exosomes are highly stable because of the protective impact of the exosomal lipid bilayers on enzymatic degradation. So the present study sought to firstly investigate the role of circulating exosomal lncRNAs in patients with AMI.

Through sequencing profiles and twice qRT‐PCR validations, circulating exosomal lncRNAs ENST00000556899.1 and ENST00000575985.1 showed significant up‐regulation in AMI. Especially ENST00000575985.1, it yielded an area under the curve value of 0.751 in AMI and was associated with many clinical parameters, including inflammatory biomarkers, prognostic indicators and myocardial damage markers, and even the risk of heart failure in AMI. ENST00000575985.1, also named as RP5‐1050D4.4‐001 (Ensembl Human GRCh37. p13) and AC004771.4‐201 (Ensembl Human GRCh38. p13), was a novel transcript antisense to calmodulin‐binding transcription factor 2 (CAMTA2) gene. No research on ENST00000575985.1 was conducted yet. However, several studies have reported the role of CAMTA2 in cardiovascular diseases.^27^, ^28^ ENST00000556899.1, which is also named as CTD‐2536I1.1‐001 (Ensembl Human GRCh37. p13) and LINC01197‐203 (Ensembl Human GRCh38. p13), was a long intergenic non‐protein coding RNA 1197. LINC01197 was reported to be associated with pancreatic cancer.^29^, ^30^ Finally, further studies are warranted to reveal the role of circulating exosomal lncRNAs ENST00000556899.1 and ENST00000575985.1 in AMI.

In summary, our findings have, for the first time, demonstrated that circulating exosomal lncRNAs ENST00000556899.1 and ENST00000575985.1 are elevated in patients with AMI, functioning as potential biomarkers for predicting the prognosis of AMI. However, the present study has some limitations: (a) as a clinical observation study, the internal mechanism of circulating exosomal lncRNAs in AMI should be further investigated; (b) included sample size in this work was small with lacked follow‐up after discharge, and thus, larger sample size study with long‐term follow‐up should be conducted to verify these findings.

## CONFLICT OF INTEREST

The authors declare that there is no conflict of interest.

## AUTHOR CONTRIBUTION

**Mei‐Li Zheng:** Data curation (supporting); Formal analysis (lead); Funding acquisition (lead); Investigation (supporting); Methodology (lead); Writing‐original draft (lead). **Xiao‐Yan Liu:** Data curation (supporting); Formal analysis (equal); Funding acquisition (supporting); Investigation (lead); Methodology (equal); Writing‐original draft (supporting); Writing‐review & editing (supporting). **Rui‐Juan Han:** Data curation (supporting); Formal analysis (supporting); Investigation (equal); Methodology (supporting); Resources (supporting); Validation (lead); Writing‐review & editing (supporting). **Wen Yuan:** Investigation (supporting); Validation (supporting). **Kai Sun:** Data curation (supporting); Resources (supporting). **Jiu‐Chang Zhong:** Conceptualization (lead); Funding acquisition (supporting); Investigation (equal); Methodology (supporting); Project administration (equal); Supervision (lead); Validation (lead); Writing‐original draft (supporting); Writing‐review & editing (lead). **Xin‐Chun Yang:** Conceptualization (lead); Data curation (lead); Formal analysis (lead); Funding acquisition (supporting); Investigation (lead); Methodology (supporting); Project administration (lead); Supervision (lead); Validation (lead); Writing‐original draft (equal); Writing‐review & editing (lead).

## Supporting information

Tables S1 and S2

## Data Availability

The data used to support the findings of this study are available from the corresponding author upon request.
