# Supplementary material for: Circulating exosomal long non‐coding RNAs in patients with acute myocardial infarction
Source: J Cell Mol Med. 2020 Jul 10;24(16):9388–96. doi: 10.1111/jcmm.15589 (PMC7417690; doi:10.1111/jcmm.15589)
Supplement: Supplementary file 1 — Tables S1 and S2 [file JCMM-24-9388-s001.docx]

Supplemental Table 1. Primers of lncRNA in real-time RT-PCR array.

| lncRNA ID  (Ensembl Human GRCh37.p13) | Forward primer (5'-3') | Reverse primer (5'-3') |
| --- | --- | --- |
| ENST00000553425.1 | CAAAGTGGTACATGGCTCATAACT | CCCGTCTCTTCCAAGGTCC |
| ENST00000556024.1 | CATTCTCACTGTGAACCCAGTTTAG | CAGACTCCGTCTGCAATCCC |
| ENST00000556899.1 | ATTACCCATCAATGAGCACCAA | GCGCACTTTCGATAAAACAGC |
| ENST00000597609.1 | ATTACCCATCAATGAGCACCAA | CGCACTTTCGATAAAACAGCTT |
| ENST00000603310.1 | TGACAAAACTACCGAACCACGTT | CATACCAAAGCAGCAGCAAAAG |
| ENST00000575985.1 | CTGGACAGGAAGAGTTGGTTAGG | TACGTAGAGAGGGCTGTCAGCC |

Supplemental Table 2. Clinical characteristics of sequencing samples from controls and patients with AMI.

|  | Control  (N=15) | AMI  (N=15) | P value |
| --- | --- | --- | --- |
| Age, years | 55.4±9.9 | 61.1±6.5 | 0.073 |
| Male, n(%) | 8 (53.3) | 12 (80.0) | 0.245 |
| Hypertension, n(%) | 7 (46.7) | 8 (53.3) | 1.000 |
| Diabetes, n(%) | 1 (6.7) | 1 (6.7) | 1.000 |
| Current smoker, n(%) | 5 (33.3) | 11 (73.3) | 0.067 |
| Current drinker, n(%) | 4 (26.7) | 6 (40.4) | 0.698 |
| Family history, n(%) | 4 (26.7) | 1 (6.7) | 0.327 |
| Heart rate, beats/min | 68.0 [60.0-77.0] | 65.0 [60.0-80.0] | 0.964 |
| Systolic blood pressure, mmHg | 123.1±34.7 | 133.7±22.8 | 0.331 |
| Diastolic blood pressure, mmHg | 74.7±8.1 | 79.9±11.5 | 0.163 |
| Body mass index, kg/m2 | 26.3±4.9 | 26.1±3.2 | 0.895 |
| Total cholesterol, mmol/L | 4.10 [3.63-5.22] | 4.75 [3.86-5.48] | 0.486 |
| HDL-C, mmol/L | 1.10 [1.00-1.59] | 1.09 [0.87-1.20] | 0.161 |
| LDL-C, mmol/L | 2.70 [1.95-3.00] | 2.83 [2.42-3.54] | 0.389 |
| Triglycerides, mmol/L | 1.12 [0.89-1.70] | 1.33 [1.11-1.88] | 0.389 |
| Fast glucose, mmol/L | 4.84 [4.39-4.98] | 6.80 [5.80-7.20] | <0.001 |

AMI, acute myocardial infarction; HDL-C, high density lipoprotein cholesterol; LDL-C, low density lipoprotein cholesterol.
